# Evaluation of the Effectiveness of the Tap Test by Combining the Use of Functional Gait Assessment and Global Rating of Change

**DOI:** 10.3389/fneur.2022.846429

**Published:** 2022-03-28

**Authors:** Masahiro Kameda, Yoshinaga Kajimoto, Yasutaka Nikaido, Akihiro Kambara, Kohei Tsujino, Hironori Yamada, Fugen Takagi, Yusuke Fukuo, Takuya Kosaka, Takuya Kanemitsu, Yoshihide Katayama, Yuichiro Tsuji, Ryokichi Yagi, Ryo Hiramatsu, Naokado Ikeda, Naosuke Nonoguchi, Motomasa Furuse, Shinji Kawabata, Toshihiro Takami, Masahiko Wanibuchi

**Affiliations:** ^1^Department of Neurosurgery, Osaka Medical and Pharmaceutical University, Takatsuki, Japan; ^2^Clinical Department of Rehabilitation, Osaka Medical and Pharmaceutical University, Takatsuki, Japan

**Keywords:** idiopathic normal pressure hydrocephalus, functional gait assessment, global rating of change scale, Timed Up and Go test, sensitivity and specificity

## Abstract

**Background:**

Although the tap test for patients with suspected idiopathic normal pressure hydrocephalus (iNPH) is still often performed as part of the preoperative evaluation, it is true that some studies have reported the limitations of the tap test, claiming that it does not provide the additional information for appropriate patient selection for surgery. We aimed to determine whether a better method of pre- and post-tap test assessment could lead to appropriate patient selection for shunting.

**Methods:**

We performed the tap test as part of the preoperative evaluation in all 40 patients who underwent lumboperitoneal shunt surgery for iNPH from April 2021 to September 2021. We retrospectively analyzed the patient data. We examined whether a comprehensive evaluation of the effect of the tap test using the Functional Gait Assessment (FGA) and Global Rating of Change (GRC) scales would identify a wider range of patients who would benefit from shunt surgery than the 3-m Timed Up and Go test (TUG) alone.

**Results:**

Assuming a prevalence of 1% for iNPH, the TUG had a sensitivity of 0.23, specificity of 0.71, positive likelihood ratio of 0.79, and negative likelihood ratio of 1.09. When improvement in either the FGA or the GRC was used as a criterion for the validity of the tap test, the sensitivity was 0.88, specificity was 0.17, positive likelihood ratio was 1.06, and negative likelihood ratio was 0.71.

**Conclusion:**

Improvement in either the FGA or the GRC is a more sensitive criterion for the effectiveness of the tap test for the gait aspect than the TUG. Since the negative likelihood ratio is lower than that for the TUG alone, it is more appropriate to exclude patients with neither FGA nor GRC improvement from surgical indications than to exclude surgical indications based on a negative TUG.

## Introduction

Idiopathic normal pressure hydrocephalus (iNPH) is a syndrome that presents as gait disturbance, incontinence, and cognitive impairment in patients with ventricle dilatation under normal cerebrospinal fluid (CSF) pressure. The causes of iNPH are unknown, and the prevalence of iNPH in Japanese residents over 65 years old is ~1.1% (1). iNPH is recognized as a treatable disease because it can be improved by shunt surgery (efficacy rate: 60–70%) (2, 3). In addition, previous reports have shown that shunt surgery for iNPH is cost-effective (4–6). To maximize the therapeutic effect of shunting for iNPH, appropriate patient selection is important.

In daily practice, patients with symptoms characteristic of iNPH, such as gait disturbance, and imaging findings characteristic of iNPH, such as Evans index >0.3 and disproportionately enlarged subarachnoid space hydrocephalus (DESH), are considered candidates for shunt surgery.

It is true that some studies have reported the limitations of the tap test, claiming that it does not provide the additional information needed to distinguish between patients who respond to shunting and those who do not (7, 8). In the Japanese Guidelines for the Management of Idiopathic Normal Pressure Hydrocephalus (3rd edition), the tap test is not always necessary anymore if there are typical imaging findings such as DESH findings. In spite of this, the tap test remains a standard preoperative evaluation method in shunt surgery. This may be because the tap test mimics shunting surgery, and confirms that symptoms will improve after the CSF is drained (1, 2, 6). Therefore, it is important to develop a method of evaluation before and after the tap test that can lead to appropriate patient selection for shunting surgery.

The 3-m Timed Up and Go test (TUG) (9) is often used to determine the effect of the tap test on the gait aspect. A previous report showed that an improvement of 5 s is a useful threshold of the TUG time at the tap test for improvement after shunt surgery, rather than the percent improvement in TUG time. However, only 37% of patients had a TUG time improvement of 5 s or more after the tap test. In addition, few patients with minor gait disturbance improved more than 5 s (10). Therefore, the TUG has the disadvantage of low sensitivity, especially in evaluating mild gait disturbance (10, 11). Several reports have attempted to analyze gait movement after the tap test in order to detect more minor changes in gait disturbance (11–17).

In this study, we report that a comprehensive evaluation of the effect of the tap test using the Functional Gait Assessment (FGA) and Global Rating of Change (GRC) scales (18, 19) can lead to more appropriate patient selection for shunting than the evaluation of the effects of TUG alone.

## Materials and Methods

### Eligible Patients

Forty patients were treated with lumboperitoneal (LP) shunt from April 2021 to September 2021 at Osaka Medical and Pharmaceutical University Hospital. Based on the Japanese iNPH guideline (1, 6), all patients underwent the tap test which involved 30 mL removal of CSF via a lumbar tap for preoperative evaluation as possible iNPH patients. All 40 patients were included in this study. This study was reviewed and approved by the ethics committee of Osaka Medical and Pharmaceutical University.

### TUG

The TUG measures, in seconds, the time taken by an individual to stand up from a standard armchair, walk a distance of 3 m, turn, walk back to the chair, and sit down again (9). TUG values after the tap test that were at least 10% less than those before the tap test were considered tap test positive. Since it has been reported that the risk of falling is higher if the TUG time is more than 13.5 s (20), patients with TUG scores <13.5 s before the tap test were classified into the “mild gait disturbance group,” and patients with TUG scores of 13.5 s or more before the tap test were classified into the “severe gait disturbance group.” In this study, the TUG was conducted as a basic evaluation method before and after the tap test to determine the effect of the tap test on the gait aspect. The TUG after the tap test was performed from immediately after the tap test until 2 weeks after the tap test. If the tap test was performed as an inpatient investigation, the TUG assessment after the tap test was mainly performed 1 or 3 days after the tap test. If the tap test was performed as an outpatient investigation, the TUG after the tap test was performed immediately after the tap test and at the next outpatient visit up to 2 weeks later. If TUG was evaluated multiple times after the tap test, the best one was used as the result of TUG after the tap test for statistical analysis. The TUG was assessed by the rehabilitation staff or the neurosurgeon.

### Functional Gait Assessment (FGA)

The Functional Gait Assessment (FGA) is a 10-item gait assessment. The FGA consists of 10 tasks, including gait level surface, change in gait speed, gait with horizontal head turns, gait with vertical head turns, gait and pivot turn, step over obstacle, gait with a narrow base of support, gait with eyes closed, ambulating backwards, and steps, and is evaluated with 0 to 3 points for each task, totaling 30 points (21). The FGA was performed as an inpatient investigation and assessed by the rehabilitation staff before and 1 or 3 days after the tap test.

### Global Rating of Change (GRC)

Following the tap test, patients were asked to indicate whether they thought there was a change in their gait using a Global Rating of Change (GRC) scale. This is a visual scale with ratings ranging from −5 to +5 whereby −5 is labeled very much worse, 0 is labeled no change, and +5 is labeled complete improvement. Patients were instructed that complete improvement means their symptoms had resolved while very much worse meant that their symptoms had become unmanageably worse (19). If the tap test was performed as an inpatient investigation, The GRC was evaluated 1 or 3 days after the tap test. If the tap test was performed as an outpatient examination, GRC assessment was performed on consecutive days after the tap test, and the mean value was calculated at the next outpatient visit to determine the efficacy. In cases where patients were unable to evaluate GRC by themselves due to cognitive problems, GRC was evaluated by their families or facility staff who were caring for them.

### INPH Grading Scale (GS)

The iNPH grading scale (GS) examines three aspects: gait disturbance, dementia, and urinary incontinence. Gait disturbance is defined as 0, normal; 1, unstable but independent gait; 2, walking with one cane; 3, walking with two canes or a walker frame; and 4, walking not possible. Dementia is defined as 0, within the normal range; 1, no apparent dementia but apathetic; 2, socially dependent but independent at home; 3, partially dependent at home; and 4, totally dependent. Urinary incontinence is defined as 0, absent; 1, absent but with pollakiuria or urinary urgency; 2, present sometimes only at night; 3, present sometimes even during the day; and 4, frequent. The grades of gait disturbance, dementia, and urinary incontinence were summated to obtain the total grade, which ranged from 0 to 12 (6). Patients were evaluated by iNPHGS before shunting and at 1, 3, and 6 months after shunting. The iNPHGS was assessed by the neurosurgeon together with the patient's interview during the consultation and the results of the TUG for the gait aspect and the MMSE for the cognitive aspect, if available.

### Measurement Parameters

We retrospectively obtained the following measurement parameters: age, sex, iNPHGS, TUG, FGA, GRC, MMSE, medical history and surgical procedure. The improvement ratio of the TUG before and after the tap test [= 1 - (TUG after tap test/TUG before tap test)], the reduction time in the TUG before and after the tap test (= TUG time before tap test—TUG time after the tap test), and the improvement score of the FGA before and after the tap test (FGA after tap test—FGA before tap test) were measured.

### LP Shunt Surgery

Patients with a positive tap test who showed some improvement in any of the three symptoms of gait, urinary incontinence, and cognitive function after the tap test were determined to be eligible for LP shunt. In addition, patients with a negative tap test but DESH findings were identified as having a high probability of iNPH and were considered eligible for LP shunt.

### Statistical Analysis

Data are presented as mean (standard deviation: SD). The sensitivity, specificity, positive likelihood ratio, and negative likelihood ratio of each assessment method as well as the combination of multiple assessment methods were examined with a prevalence of iNPH as 1% (1). Spearman correlation was utilized to calculate the relationships among the improvement ratio of the TUG, the improvement score of the FGA, and the GRC score. Data analyses were performed using JMP 10 or IBM SPSS ver28.

## Results

### Overall Results

The 40 patients consisted of 21 males and 19 females. The mean age at surgery was 78.8 years (SD 5.2). The most common background of patients was underlying hypertension. Some patients were on medical therapy for Alzheimer's disease or dementia with Lewy bodies (DLB), and 3 patients were transitioned from asymptomatic ventriculomegaly with features of iNPH on MRI (AVIM) to iNPH (Table 1).

**Table 1:** Patient characteristics (*n* = 40).

**Age**	**78.8 (SD 5.2)**	
Sex	Male, Female (19, 21)	
Hypertension (*n*, %)	19, 47.5	
Hyperlipidemia (*n*, %)	14, 30	
Diabetes mellitus (*n*, %)	5, 12.5	
Dementia with Lewy bodies (*n*, %)	1, 2.5	
Alzheimer's disease (*n*, %)	6, 15	
Transition to iNPH after AVIM (*n*, %)	3, 7.5	
**Pre tap test evaluation**		
	TUG	15.3 (SD 5.7)
	FGA	17.3 (SD 5.8)
	MMSE	24.5 (SD 4.6)
**Post tap test evaluation**		
	TUG	14.2 (SD 5.1)
	FGA	20.2 (SD 5.6)
	GRC for gait aspect	1.5 (SD 1.3)
	MMSE	24.5 (SD 4.9)
Preoperative iNPHGS (total)	5.2 (SD 2.4)	
Preoperative iNPHGS for gait aspect	1.8 (SD 0.9)	
Postoperative iNPHGS (total)	3.4 (SD 2.4)	
Postoperative iNPHGS for gait aspect	1.2 (SD 1.0)	
TUG after shunt surgery	12.8 (SD 4.6)	
MMSE after shunt surgery	25.5 (SD 4.3)	

Due to the prevalence of COVID-19, the tap test could not be performed as an inpatient investigation in all patients, which is why homogeneous preoperative evaluation could not be performed. However, despite the differences between inpatient and outpatient investigations, all patients were evaluated for gait disturbance by TUG, although there were variations in the timing of evaluation after the tap test. As a result, FGA was performed in 24 patients and GRC in 28 patients. The effectiveness of the tap test was determined on the basis of the TUG and also by taking into account the FGA and GRC data collected for some patients.

As for the FGA score, 20 patients (83%) showed an improvement of 1 or more after the tap test. As for the GRC, 20 patients (71%) reported an improvement in gait disturbance after the tap test.

Thirty-seven patients had a positive tap test and exhibited some improvement in gait, urinary, or cognitive function symptoms, and three patients had a negative tap test but disproportionately enlarged subarachnoid space hydrocephalus on magnetic resonance imaging. These 40 patients were treated with LP shunt as probable iNPH patients. Subsequently, 32 patients (80%) had improvement in the iNPHGS after shunting and were diagnosed with definite iNPH.

The forty patients in this study were divided into the severe gait disturbance group (19 patients; 47.5%) and the mild gait disturbance group (21 patients; 52.5%), in which the TUG before the tap test was 13.5 s or more and <13.5 s, respectively. Of these 40 patients, only 10 (25%) showed an improvement of 10% or more in the TUG. Only one of these 10 patients (10%) was in the mild gait disorder group. Although three of the 40 patients (8%) had an improvement of 5 s or more in the TUG, none of them were in the mild gait disorder group. Although cutoff values such as an improvement ratio of the TUG by more than 10% or a reduction in TUG time by more than 5 s are often used to judge the effect of the tap test, these cutoff values were found to be less sensitive, especially in the mild gait disturbance group.

### TUG vs. FGA

In addition to the TUG, 24 patients were also evaluated by FGA before and after the tap test to determine the effect of the tap test on the gait aspect. Based on the TUG before the tap test, 10 patients (42%) were included in the severe gait disturbance group and 14 patients (58%) were included in the mild gait disturbance group. Four patients (17%) showed a more than 10% improvement in the TUG, and all of them were in the severe gait disorder group. In addition, one patient (4%) showed an improvement of more than 5 s in the TUG, and this patient was in the severe gait disturbance group. The improvement ratio of the TUG after the tap test in the mild gait disturbance group was 4.4%, and it was thus considered difficult to find an improvement that exceeds these cutoff values (reduction of TUG time by more than 10% or more than 5 s) when the gait disturbance is mild.

In contrast, for the FGA, 20 of 24 patients (83%) showed FGA improvement of 1 or more after the tap test. Eighteen patients (75%) improved the gait aspect of the iNPHGS after shunt surgery. In the group of 14 patients with mild gait disturbance, 12 patients (86%) had an FGA improvement of 1 or more after the tap test, and 10 patients (71%) improved their gait aspect of the iNPHGS after shunt surgery. Therefore, FGA appeared to be more useful than the TUG in identifying patients who would respond to shunting.

The indications for shunt surgery were determined by referring to the improvement of other parameters, such as urinary incontinence and cognitive function as well as gait function after the tap test. A total of 21 of the 24 patients (88%) who were evaluated by the FGA and TUG had an improvement in the total score of the three parameters of the iNPHGS after shunt surgery.

#### Sensitivity Specificity, Positive Likelihood Ratio, and Negative Likelihood Ratio of the TUG, FGA, and GRC for Improvement of Gait Score in the Post-shunt INPHGS

In 26 out of 40 patients, an improvement of the gait score in the iNHPGS was observed.

### TUG

Of the 10 patients who showed an improvement of 10% or more in the TUG, six showed improvement of gait score in the post-shunt iNHPGS. Of the 30 patients who did not show an improvement of 10% or more in the TUG, 20 showed improvement of gait score in the post-shunt iNPHGS. The sensitivity and specificity were thus 0.23 [95%CI: 0.14–0.32] and 0.71 [95%CI: 0.54–0.87], respectively. Based on a prevalence of 1% for iNPH, the positive likelihood ratio was 0.79 and the negative likelihood ratio was 1.09 (Table 2).

**Table 2:** Sensitivity specificity, positive likelihood ratio, and negative likelihood ratio of the TUG, FGA, and GRC for improvement of gait score in the post-shunt iNPHGS.

	**Sensitivity**	**Specificity**	**Positive likelihood ratio**	**Negative likelihood ratio**
TUG	0.23	0.71	0.79	1.09
FGA	0.83	0.17	1.00	1.00
GRC	0.70	0.25	0.93	1.20

### FGA

Of the 20 patients who showed improvement by the FGA, 15 also showed improvement by the iNHPGS. Of the four patients who did not show improvement by the FGA, three showed improvement by the iNPHGS. The sensitivity and specificity were thus 0.83 [95%CI: 0.79–0.92] and 0.17 [95%CI: 0.03–0.44], respectively. Based on a prevalence of 1% for iNPH, the positive likelihood ratio was 1.00 and the negative likelihood ratio was 1.00.

### GRC

Of the 20 patients who showed improvement by the GRC, 14 showed improvement of gait score in the post-shunt iNHPGS. Of the eight patients who did not show improvement by the GRC, six showed improvement of gait score in the post-shunt iNPHGS. The sensitivity and specificity were thus 0.70 [95%CI: 0.63–0.81] and 0.25 [95%CI: 0.08–0.52], respectively. Based on a prevalence of 1% for iNPH, the positive likelihood ratio was 0.93 and the negative likelihood ratio was 1.20.

#### Sensitivity Specificity, Positive Likelihood Ratio, and Negative Likelihood Ratio of the FGA, and GRC for Improvement of Gait Score in the Post-shunt INPHGS in the Mild Gait Disturbance Group

As for the 14 patients in the mild gait disturbance group, of the 12 patients who showed improvement by the FGA, 8 showed improvement by the iNHPGS. Of the two patients who did not show improvement by the FGA, both showed improvement by the iNPHGS. The sensitivity and specificity were thus 0.80 [95%CI: 0.80–0.92] and 0 [95%CI: 0.00–0.31], respectively. Based on a prevalence of 1% for iNPH, the positive likelihood ratio was 0.80 and the negative likelihood ratio was infinite (Table 3).

**Table 3:** Sensitivity specificity, positive likelihood ratio, and negative likelihood ratio of the FGA, and GRC for improvement of gait score in the post-shunt iNPHGS in the mild gait disturbance group.

	**Sensitivity**	**Specificity**	**Positive likelihood ratio**	**Negative likelihood ratio**
FGA	0.80	0.00	0.80	Infinite
GRC	0.64	0.20	0.80	1.80

As for the 16 patients in the mild gait disturbance group, of the 11 patients who showed improvement by the GRC, 7 showed improvement by the iNHPGS. Of the 5 patients who did not show improvement by the GRC, 4 showed improvement by the iNPHGS. Therefore, the sensitivity and specificity were 0.64 [95%CI: 0.56–0.79] and 0.20 [95%CI: 0.04–0.54], respectively. Based on a prevalence of 1% for iNPH, the positive likelihood ratio was 0.80 and the negative likelihood ratio was 1.80.

As shown above, all indices were worse when only the mild gait disturbance group was evaluated as compared to when all 40 patients were evaluated.

### Evaluation Method for the Effectiveness of the Tap Test Combined With the FGA and GRC

The GRC score exhibited the strongest correlation with the improvement score of the FGA (r = 0.577), followed by the improvement ratio of TUG time (r = 0.401) whereas the improvement score of the FGA and the improvement ratio of TUG time exhibited almost no correlation (r = 0.110).

**Figure 1:**
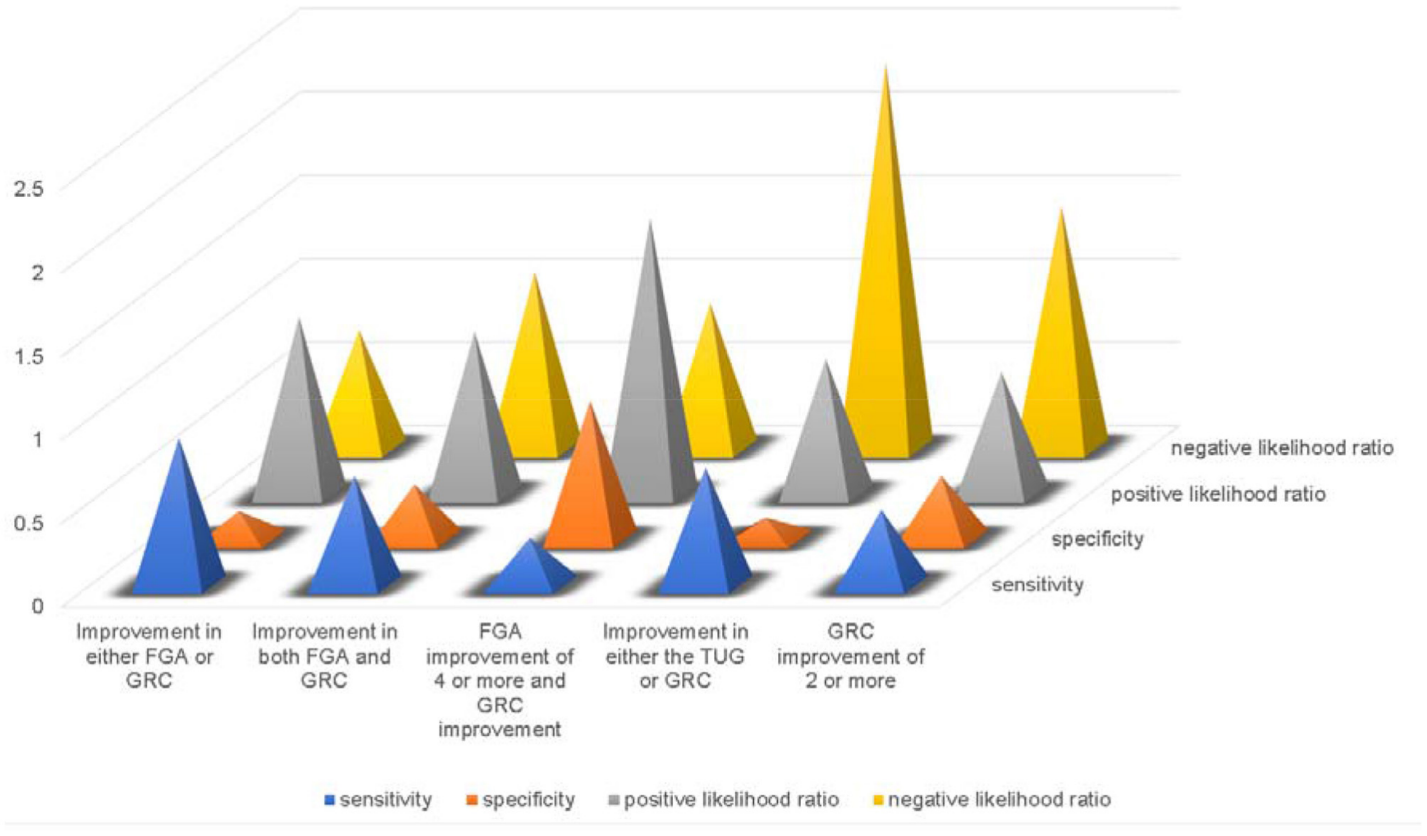
Comparison of evaluation methods for the effectiveness of the tap test.

We investigated whether the combination of the FGA and GRC as well as the TUG could be used to identify patients who would benefit from shunt surgery but who could not be picked up by the TUG, and to determine the effectiveness of the tap test (Figure 1).

#### Improvement in Either FGA or GRC

Among the 23 patients with both FGA and GRC data, 20 (87%) showed improvement in either the FGA or GRC, and the iNPHGS gait scale improved in 15 patients (75%). In the remaining three patients, the iNPHGS gait scale improved in two patients. The sensitivity and specificity were thus 0.88 [95%CI: 0.84–0.96] and 0.17 [95%CI: 0.03–0.39], respectively. Based on a prevalence of 1% for iNPH, the positive likelihood ratio was 1.06 and the negative likelihood ratio was 0.71.

#### Improvement in Both FGA and GRC

Among the 23 patients for whom both FGA and GRC data were available, 19 patients showed an improvement of at least 1 in the FGA, and 15 of them showed improvement in gait disturbance in the GRC. Of the 15 patients who showed improvement in both the FGA and GRC, 11 (73%) showed improvement in the iNPHGS. Using this entry criterion (improvement in both FGA and GRC), eight patients were tap-negative, and 6 of these patients (75%) showed improvement in the iNPHGS. The sensitivity and specificity were thus 0.65 [95%CI: 0.57–0.76] and 0.33 [95%CI: 0.10–0.65], respectively. Based on a prevalence of 1% for iNPH, the positive likelihood ratio was 0.97 and the negative likelihood ratio was 1.06.

#### FGA Improvement of 4 or More

Patients who did not show improvement in the GRC showed an improvement of 3 or less in the FGA. In contrast, all patients with FGA improvement of 4 or more also reported improvement in the GRC, but only 6 (25%) of 24 patients had FGA improvement of 4 or more. Among the six patients with FGA improvement of 4 or more (all of which had GRC improvement), five (83%) showed improvement in the iNPHGS. Using this entry criterion (FGA improvement of 4 or more and GRC improvement), 18 patients were tap-negative, and 13 of these patients (72%) showed improvement in the iNPHGS. The sensitivity and specificity were thus 0.28 [95%CI: 0.17–0.32] and 0.83 [95%CI: 0.52–0.97], respectively. Based on a prevalence of 1% for iNPH, the positive likelihood ratio was 1.65 and the negative likelihood ratio was 0.87.

#### Improvement in Either the TUG or GRC

Twenty-one patients had an improved TUG or GRC, and 14 of them (66%) had an improved iNPHGS gait scale. Of the seven patients who did not show any improvement, six improved their iNPHGS gait scale. The sensitivity and specificity were thus 0.70 [95%CI: 0.66–0.81] and 0.13 [95%CI: 0.02–0.39], respectively. Based on a prevalence of 1% for iNPH, the positive likelihood ratio was 0.81 and the negative likelihood ratio was 2.31.

#### GRC Improvement of 2 or More

When the GRC score of 2 or more was judged to be positive for the tap test, 14 of 28 patients were positive for tap, and 9 of them improved their iNPHGS gait scale. A total of 14 patients were negative for tap, and 11 of them improved their iNPHGS gait scale. The sensitivity and specificity were thus 0.45 [95%CI: 0.36–0.56] and 0.38 [95%CI: 0.15–0.66], respectively. Based on a prevalence of 1% for iNPH, the positive likelihood ratio was 0.73 and the negative likelihood ratio was 1.45.

## Discussion

Idiopathic normal pressure hydrocephalus (iNPH) is a very complicated and easily misdiagnosed disease. Various studies have been conducted to determine which patients would benefit from surgery.

The tap test seems to be an attractive evaluation method because it actually mimics LP shunt surgery, as the spinal fluid is removed by lumbar puncture.

However, it has been reported that the tap test does not provide the necessary information to distinguish between patients who respond to shunting and those who do not (7), and this has led to research on the development of evaluation methods other than the tap test (22) or the combination of other tests in addition to the tap test (8) for more appropriate patient selection. In addition, there have been studies on what kind of gait evaluation method is effective after the tap test and the appropriate timing of evaluation after the tap test. It has been reported that the time and the number of steps taken in a 10 m walk at free speed after the tap test correlate with the improvement of iNPH grading scale (23). Other reports suggest that the Tinetti Tool Assessment which involves different gait parameters is more effective at 72 h than at 24 h as the timing of evaluation after the tap test (24, 25).

In this study, we conducted TUG in all patients, which is the standard evaluation method in Japan. The disadvantage of TUG is its low sensitivity (26). Although cutoff values such as a reduction in time by more than 10% or a reduction in time by more than 5 s are often used to judge the effect, it is difficult to find an improvement that exceeds these cutoff values after the tap test when the gait disturbance is mild.

Our team has previously reported that the FGA score is an independent factor associated with the risk of falls associated with iNPH (17). In addition, we reported that the FGA may be suitable for distinguishing patients with mild iNPH who are more likely to fall from those who are less likely to fall (27), and that the FGA score can provide a more detailed representation of improvement after the tap test than the TUG, even in patients with mild gait disturbance (16).

Recently, there have been reports on the use of the GRC by patients or their families in addition to the TUG, Performance Oriented Mobility Assessment (Tinetti), and Berg Balance Scale (BBS) by medical professionals to determine the efficacy after the tap test (19, 28) or shunt surgery (29). Therefore, in this study, we investigated whether the FGA and GRC, as well as the TUG, could be combined to identify patients who are likely to benefit from shunt surgery but cannot be picked up by the TUG, and to determine the validity of the tap test.

It has been reported that the minimum detectable change of the GRC is 0.45 on an 11-point scale (18, 30). It has also been reported that the minimal clinically important change, which is the change most likely to be relevant to patients, is approximately half the standard deviation of the outcome measures (31). In the present study, the improvement in the FGA score (*n* = 28) was 2.78 (SD 1.93) and that in the GRC (*n* = 23) was 1.48 (SD 1.27). Thus, the minimally important differences (minimal clinically important change and minimal clinically important difference) of the improvement of the FGA score and GRC were 0.97 and 0.64, half of the SDs, respectively. Therefore, an improvement of 1 point or more in either the FGA or GRC is clinically meaningful and important. In addition, our group reported that, in order to produce an improvement of more than 2 points in the GRC after shunt surgery, the postoperative FGA needs to improve by more than 4 points compared to the preoperative FGA (16). Therefore, the sensitivity, specificity, positive likelihood ratio, and negative likelihood ratio were also calculated for cutoff values, such as an improvement of GRC 2 points or more in addition to an improvement of GRC 1 point or more, and an improvement of FGA 4 points or more in addition to an improvement of FGA 1 point or more.

The higher the sensitivity or specificity, the larger the value of the positive likelihood ratio and the smaller the value of the negative likelihood ratio. The larger the value of the positive likelihood ratio and the smaller the value of the negative likelihood ratio, the more useful they are as a criterion. As shown in section Sensitivity Specificity, Positive Likelihood Ratio, and Negative Likelihood Ratio of the TUG, FGA, and GRC for Improvement of Gait Score in the Post-Shunt iNPHGS, the TUG had a sensitivity of 0.23, specificity of 0.71, positive likelihood ratio of 0.79, and negative likelihood ratio of 1.09. In the present study, we calculated the sensitivity, specificity, positive likelihood ratio, and negative likelihood ratio of various cutoff criteria by combining not only the TUG but also the FGA and GRC, and found no new criterion that was better than the TUG in both sensitivity and specificity. There were no new criteria that were superior to the TUG not only in sensitivity and specificity, but also in positive and negative likelihood ratios when calculated with a prevalence of 1%. Most of the cutoff criteria examined in this study were inferior to the TUG in all indices. However, when improvement in either the FGA or GRC was considered as a criterion for the effectiveness of the tap test, the sensitivity was 0.88, specificity was 0.17, positive likelihood ratio was 1.06, and negative likelihood ratio was 0.71, as shown in section Improvement in Either FGA or GRC. We thought that this criterion might be a candidate as an alternative cutoff criterion to the TUG.

When this criterion was used as the criterion for evaluating the effectiveness of the tap test, it was confirmed that the negative likelihood ratio was lower than that of the TUG alone. Therefore, it is more appropriate to exclude patients who do not show improvement in either the FGA or GRC. In addition, when improvement in either the FGA or GRC is used as a criterion for judging the efficacy of the tap test for the gait aspect, the sensitivity of the test is higher than that of the TUG alone, although the specificity is lower. This high sensitivity but low specificity may mean that there will be more patients who undergo surgery but do not benefit from the treatment. Nevertheless, in the present study, only 25% of patients were tap test positive when the TUG alone was used for patient selection, but with this criterion (improvement in either the FGA or GRC), the number of tap test–positive patients increased to 87%. Seventy-five percent of these patients improved in the iNPHGS gait scale with shunt surgery. In clinical practice, the effectiveness of the tap test is judged together with the improvement of symptoms other than gait, but we believe that excluding patients from shunting because of a negative TUG result, at least when the gait is evaluated only by TUG, does not adequately identify patients who would benefit from shunting.

One limitation of this study is that due to the prevalence of covid-19, preoperative evaluations were mixed between those performed in the inpatient setting and those performed in the outpatient setting, resulting in variations in the timing and method of evaluation. Another limitation is that the number of cases was not large and this was a retrospective study because all surgeries were performed at one institution during 6 months.

## Conclusion

Improvement in either the FGA or GRC is a more sensitive criterion for the efficacy of the tap test for the gait aspect than the TUG. Since the negative likelihood ratio is lower than that of the TUG alone, it is more appropriate to exclude surgical indications in patients with neither FGA nor GRC improvement than to exclude surgical indications with no improvement in the TUG.

## Data Availability Statement

The raw data supporting the conclusions of this article will be made available by the authors, without undue reservation.

## Ethics Statement

The studies involving human participants were reviewed and approved by Osaka Medical and Pharmaceutical University. Written informed consent for participation was not required for this study in accordance with the national legislation and the institutional requirements.

## Author Contributions

MK, YK, and YN made substantial contributions to the conception and design of the study. All authors contributed to the acquisition, analysis, or interpretation of data for the study by patient management. MK wrote the draft. All authors revised the manuscript with critical comments and approved the final version.

## Funding

This work was supported by Japan Society for the Promotion of Science Grant-in-Aid for Scientific Research 19K09528, 19K10405, 20K09390.

## Conflict of Interest

The authors declare that the research was conducted in the absence of any commercial or financial relationships that could be construed as a potential conflict of interest.

## Publisher's Note

All claims expressed in this article are solely those of the authors and do not necessarily represent those of their affiliated organizations, or those of the publisher, the editors and the reviewers. Any product that may be evaluated in this article, or claim that may be made by its manufacturer, is not guaranteed or endorsed by the publisher.
