# Corrigendum: Evaluation of the Effectiveness of the Tap Test by Combining the Use of Functional Gait Assessment and Global Rating of Change

**DOI:** 10.3389/fneur.2022.926924

**Published:** 2022-05-30

**Authors:** Masahiro Kameda, Yoshinaga Kajimoto, Yasutaka Nikaido, Akihiro Kambara, Kohei Tsujino, Hironori Yamada, Fugen Takagi, Yusuke Fukuo, Takuya Kosaka, Takuya Kanemitsu, Yoshihide Katayama, Yuichiro Tsuji, Ryokichi Yagi, Ryo Hiramatsu, Naokado Ikeda, Naosuke Nonoguchi, Motomasa Furuse, Shinji Kawabata, Toshihiro Takami, Masahiko Wanibuchi

**Affiliations:** ^1^Department of Neurosurgery, Osaka Medical and Pharmaceutical University, Takatsuki, Japan; ^2^Clinical Department of Rehabilitation, Osaka Medical and Pharmaceutical University, Takatsuki, Japan

**Keywords:** idiopathic normal pressure hydrocephalus, functional gait assessment, global rating of change scale, Timed Up and Go test, sensitivity and specificity

“Yasutaka Nikaido” was mistakenly not included as an author in the published article. The corrected “**Author Contributions**” statement appears below. A new affiliation has also been included for this author—affiliation 2, “Clinical Department of Rehabilitation, Osaka Medical and Pharmaceutical University, Takatsuki, Japan.”

## Author Contributions

MK, YK, and YN made substantial contributions to the conception and design of the study. All authors contributed to the acquisition, analysis, or interpretation of data for the study by patient management. MK wrote the draft. All authors revised the manuscript with critical comments and approved the final version.

The authors apologize for this error and state that this does not change the scientific conclusions of the article in any way. The original article has been updated.

## Publisher's Note

All claims expressed in this article are solely those of the authors and do not necessarily represent those of their affiliated organizations, or those of the publisher, the editors and the reviewers. Any product that may be evaluated in this article, or claim that may be made by its manufacturer, is not guaranteed or endorsed by the publisher.

